# Multiple G-quartet structures in pre-edited mRNAs suggest evolutionary driving force for RNA editing in trypanosomes

**DOI:** 10.1038/srep29810

**Published:** 2016-07-20

**Authors:** W.-Matthias Leeder, Niklas F. C. Hummel, H. Ulrich Göringer

**Affiliations:** 1 Molecular Genetics, Darmstadt University of Technology, Schnittspahnstraße 10, 64287 Darmstadt, Germany

## Abstract

Mitochondrial transcript maturation in African trypanosomes requires a U-nucleotide specific RNA editing reaction. In its most extreme form hundreds of U’s are inserted into and deleted from primary transcripts to generate functional mRNAs. Unfortunately, both origin and biological role of the process have remained enigmatic. Here we report a so far unrecognized structural feature of pre-edited mRNAs. We demonstrate that the cryptic pre-mRNAs contain numerous clustered G-nt, which fold into G-quadruplex (GQ) structures. We identified 27 GQ’s in the different pre-mRNAs and demonstrate a positive correlation between the steady state abundance of guide (g)RNAs and the sequence position of GQ-elements. We postulate that the driving force for selecting G-rich sequences lies in the formation of DNA/RNA hybrid G-quadruplex (HQ) structures between the pre-edited transcripts and the non-template strands of mitochondrial DNA. HQ’s are transcription termination/replication initiation sites and thus guarantee an unperturbed replication of the mt-genome. This is of special importance in the insect-stage of the parasite. In the transcription-on state, the identified GQ’s require editing as a GQ-resolving activity indicating a link between replication, transcription and RNA editing. We propose that the different processes have coevolved and suggest the parasite life-cycle and the single mitochondrion as evolutionary driving forces.

African trypanosomes are single cell blood-parasites and as such they are of medical importance^1^. The parasites go through a life cycle that involves a mammalian host and tsetse flies as transmitting vectors. Differentiation between the two life cycle stages is accompanied by considerable metabolic changes and specifically in the tsetse fly the parasites require fully functional mitochondria to synthesize ATP. Importantly, trypanosomes have only one mitochondrion per cell (Fig. 1a). As a consequence, the precise duplication and inheritance of the mitochondrial genome is essential for the parasite to maintain its life cycle^2^. The mitochondrial genome, also known as kinetoplastid (k)DNA, is organized as a macromolecular network of two types of interlocked circular DNA molecules: a heterogenous population of several thousand 1 kb-size, guide (g)RNA-encoding minicircles and ≤50 identical copies of so-called maxicircles^3^. Each maxicircle has a molecular size of 23 kbp. It carries the genetic information for two ribosomal RNAs (9S, 12S), one ribosomal protein (S12) and seventeen additional protein-coding sequences (Fig. 1c). Sequence conservation of the predicted polypeptides indicates that they represent a subset of subunits of the electron transport and oxidative phosphorylation systems. Importantly, 12 of the 18 open reading frames (ORF) require RNA editing in order to be converted into translatable mRNAs^4^,^5^,^6^. For nine of these ORF’s this involves the site-specific insertion and deletion of literally hundreds of U-nucleotides, a phenomenon that has been dubbed pan-editing^7^. Pan-editing increases the U-nucleotide content of the different transcripts on average from 27% to almost 60% (Fig. 1b). While the change in the U-nt content of the pre-edited mRNAs is without precedence, it has resulted in a U-centric perspective on the phenomenon (Fig. 1d). However, alternative perceptions are possible.

Here we advocate for a G-nt focussed viewpoint (Fig. 1e). By analyzing the nucleotide propensity of all pre-edited mitochondrial mRNAs in *Trypanosoma brucei* we identified an extreme purine nucleotide bias in all pan-edited pre-mRNAs and we demonstrate that specifically G-nucleotides are arranged in tracts of G’s. G-rich DNA and RNA sequences have the ability to fold into thermodynamically very stable, non-canonical, higher-order structures known as G-quadruplex (GQ)-folds^8^. They consist of multiple (≥2) stacked arrays of four Hoogsteen-bonded guanosine nucleotides (G-tetrads) that are stabilized by potassium cations. Potential GQ-forming RNA and DNA sequences have been identified in many species ranging from bacteria to animals and have been implicated in a variety of phenomena including genome instability, telomere maintenance and the initiation of DNA-replication^8^,^9^. As a consequence of the high number of repetetive G-runs in the pre-edited mRNAs of *T. brucei* we analyzed whether GQ-elements can be identified in these RNAs using a combination of bioinformatic search algorithms and a reverse transcriptase stop assay. We demonstrate that all but one of the pan-edited pre-mRNAs contain multiple, up to five GQ-folds. We further demonstrate that RNA editing progressively resolves the different GQ-elements and that the positions of the various GQ’s correlate with the steady state abundance of gRNAs. In analogy to other biological systems we hypothesize that the GQ-elements function to separate mitochondrial transcription from mitochondrial DNA-synthesis, which provides a new conceptual framework for the evolutionary driving force(s) of RNA editing in African trypanosomes and other kinetoplastid organisms.

## Results

### G-nucleotide cluster analysis of pre-edited mRNAs

**Figure 1:**
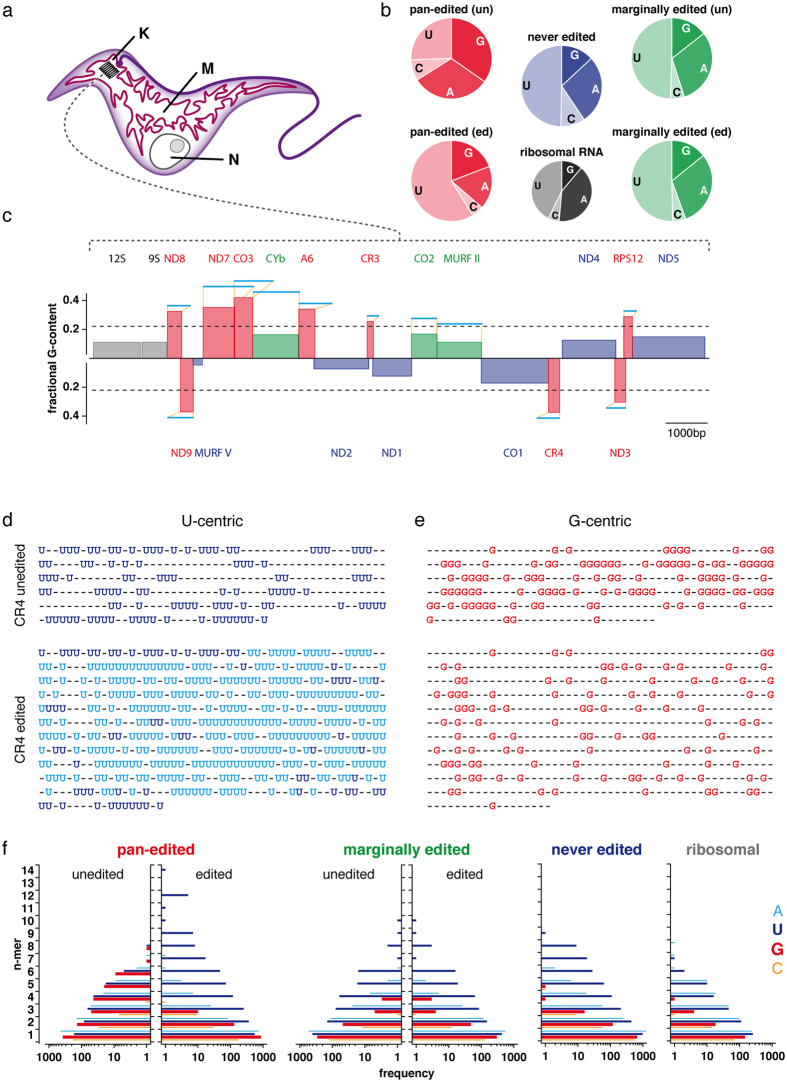
*T. brucei* mitochondrial genome organization and nucleotide propensities. (**a**) Sketch of an insect-stage *T. brucei* cell highlighting the single, extended tubular mitochondrion (M) in maroon. N: nucleus; K: kinetoplast mitochondrial DNA. (**b**) Nucleotide content of the different classes of mitochondrial transcripts. Red: pan-edited RNAs in their unedited (un) and edited (ed) state. Green: marginally edited RNAs in their unedited (un) and edited (ed) state. Blue: never-edited transcripts. Grey: ribosomal RNAs. (**c**) Linear map of both strands of the coding region of the *T. brucei* mitochondrial DNA maxicircle. Genes are annotated as boxes and are colored as in (**b**). Ribosomal RNA genes: 9S, 12S; pan-edited genes: ND8, ND7, CO3, A6, CR3, RPS12, ND3, CR4, ND9; marginally edited genes: CYb, CO2, MURF II; never-edited genes: ND4, ND5, CO1, ND1, ND2, MURF V. The height of the individual boxes indicates the fractional G-content of the different ORF’s. Dashed line: average G-content of 22%. All pan-edited genes are above the mean. Lines in light blue above the individual genes indicate the nt-length of the fully edited transcripts (for details see Supplementary Table 1). (**d**) “U-centric” view of the unedited (top) and edited (bottom) transcript of CR4. Dark blue: U-nucleotides in the pre-edited mRNA. Light blue: Inserted U’s as a result of RNA editing. (**e**) “G-centric” view of the unedited (top) and edited (bottom) CR4-transcript emphasizing the conversion from a high number of G-runs (2 ≤ G ≤ 6) in the unedited state to a low number of G-tracts in the edited state. (**f**) Homopolymer cluster (n-mer)-analysis of all pan-edited, marginally edited and never-edited RNAs in *T. brucei* (Light blue: A-nt, dark blue: U-nt, red: G-nt, orange: C-nt). The editing-mediated reduction of homopolymer runs is unique to G-nt.

Pan-edited pre-mRNAs in their unedited state lack substantial nucleotide information (on average 45%). Furthermore, they are characterized by an unusual high G-content. On average 34% of the nucleotides are G’s (Fig. 1b; Supplementary Table 1). As a consequence the purin/pyrimidine (R/Y) ratio of the transcripts varies between 1.5 ≤ R/Y ≤ 2.7 with a mean of R/Y = 2. Editing reduces the G-content to an average of 19% and a mean R/Y of 0.6. Moreover, the different pre-edited transcripts contain high numbers of tracts of G-nucleotides (2 ≤ G ≤ 8): 67% of all G’s are arranged in clusters and editing reduces the number of G-runs to 25% (Fig. 1f). This is a unique feature of G-nucleotides in these transcripts and neither A’s nor C’s show the same characteristic. Especially A-nucleotides, which are clustered to a similar degree do not become resolved. As such the editing reaction can be viewed as a process specifically resolving the G-cluster propensity of the different pre-edited mRNAs (Fig. 1e,f).

### Pre-edited transcripts contain multiple GQ-elements

**Figure 2:**
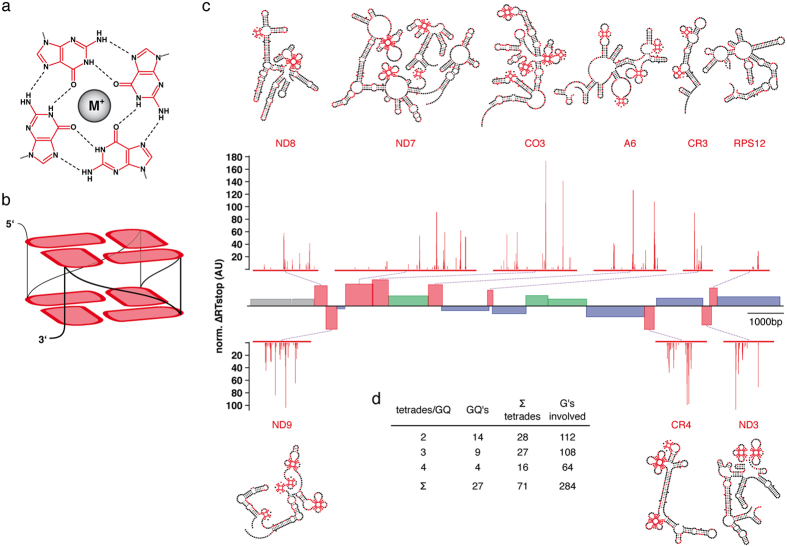
Experimental identification of GQ-folds. (**a**) Structure of a G-tetrade consisting of four Hoogsteen-bonded (black dashed lines) G-residues (red) coordinated by a stabilizing monovalent cation (M^+^). (**b**) Parallel, intramolecular G-quadruplex formed by two stacked G-tetrades. Black line: phosphate/sugar backbone. Loop nucleotides are not shown for clarity. (**c**) Differential normalized reverse transcriptase (norm. ΔRT)-stop profiles (red) and predicted minimal free energy (MFE)-2D-structures of all pan-edited *T. brucei* transcripts in their pre-edited folding state. The individual structures are shown above and below the linear map of the coding region of the *T. brucei* maxicircle DNA as introduced in Fig. 1. Grey: ribosomal RNA genes; red: pan-edited genes; green: marginally edited genes; blue: never-edited genes. GQ-folds are drawn as “leaf-like” structures in red. AU: arbitrary unit. (**d**) Summary of the characteristics of all GQ-elements in the pan-edited transcripts.

G-rich DNA and RNA sequences have been shown to adopt thermodynamically highly stable, non-canonical, higher-order structures termed G-quadruplex (GQ)-elements (Fig. 2a,b). They consist of a minimum of two stacked arrays of four Hoogsteen-bonded G-nucleotides (G-tetrads) that are stabilized by monovalent cations such as potassium. Based on the above identified high number of clustered G’s in the pan-edited transcripts we analyzed both strands of the of *T. brucei* maxicircle for putative GQ-forming sequences using predictive algorithms. Supplementary Fig. 2 summarizes the result of a QGRS (Quadruplex-forming-G-Rich-Sequences) Mapper analysis^10^ using the generic GQ-motif G_>2_N_y1_G_>2_N_y2_G_>2_N_y3_G_>2_. Strikingly, we identified a very high number of putative GQ-forming G-nt almost exclusively in the nine pan-edited transcripts (ND8, ND9, ND7, CO3, A6, CR3, CR4, ND3 and RPS12). The same result was obtained using an energy function-based RNA-folding algorithm (ViennaRNA^11^) and was further supported by analyzing the mitochondrial transcriptomes of the related kinetoplastid organisms *Trypanosoma cruzi* and *Leishmania tarentolae* (Supplementary Fig. 3). Although the extent of editing is lower in *L. tarentolae*, potential GQ-forming G-nt exclusively map to the pan-edited transcripts ND8, ND9, CR3, CR4, ND3 and RPS12.

Due to the inherent possibility of false-positive and false-negative predictions we went on and identified the number of GQ-folds by experimental means. For that we used a reverse transcriptase (RT) stop assay^12^. In conjunction with DNA-sequencing the assay enables the positional mapping of GQ-folds with nucleotide resolution. As an additional criterion we monitored the suppression of the different RT-stop signals in the presence of Na^+^- and Li^+^ -ions since G-quartets fold in a cation-dependent manner (K^+^ ≫ Na^+^>Li^+^). Figure 2c shows a summary of the experiments. *In toto* we mapped 27 GQ-folds in the 9 pan-edited transcripts. With the exception of the RPS12 transcript^13^ all pre-mRNAs contain minimally 2 GQ’s with an average of 3.0 GQ’s/pre-mRNA. The highest number was identified in the ND9- and CO3-transcripts, which hold 5 GQ’s. The 27 GQ’s involve a total of 284 G-nt and form 71 G-tetrades. This calculates to an average of 2.6 *i.e.* 3 G-tetrades/GQ-fold (Fig. 2d). Almost all RT-stop profiles are in line with the presence of ensembles of GQ-containing 2D-structures involving different G-nt within the clustered G-runs^14^. In some cases mutually exclusive GQ’s exist (Supplementary Fig. 4).

### RNA editing is a GQ-fold resolving process

**Figure 3:**
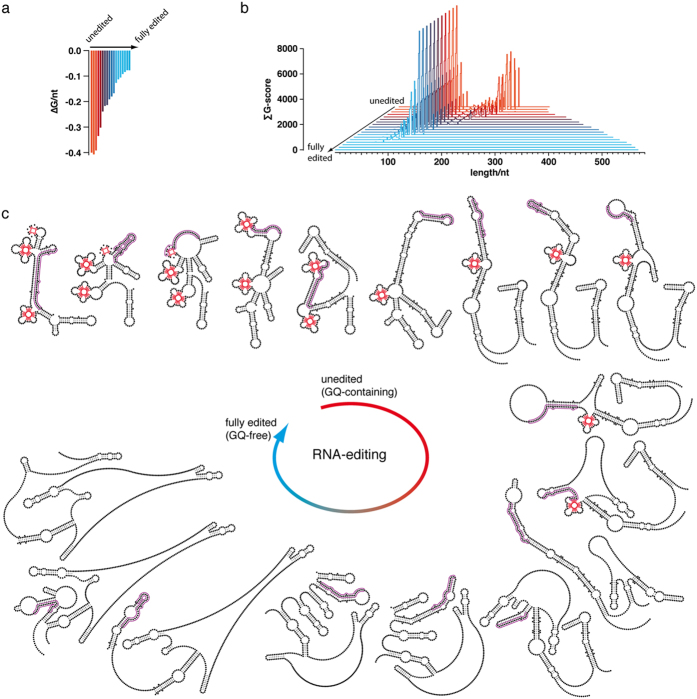
RNA editing as a GQ-resolving process. Gibbs free energy (ΔG/nt) changes (**a**) and cumulative G-score (∑G) changes (**b**) of the CR4-transcript over the course of seventeen gRNA-driven reaction steps. The colour transition from red to blue annotates the stepwise decrease in the number of G-tracts or inversely the stepwise increase in the U-content of the CR4-RNA. (**c**) MFE-2D-structures of the CR4-transcript from its unedited to its fully edited folding state (clockwise starting in the upper left corner). Unedited CR4-RNA contains 3 GQ-elements (“leaf-like” structures in red), which are progressively resolved to generate a fully edited, GQ-free mRNA. Guide RNA interaction sites are encircled in pink.

As a next step we analyzed the structural fate of the different GQ’s as a consequence of editing. For that we calculated the G-score- and 2D-structure-changes of the different transcripts over the course of all gRNA-directed editing steps. Figure 3 shows as an example the analysis of the CR4-transcript. In its pre-edited form the pre-mRNA contains 3 GQ’s and requires 17 gRNAs to become fully edited. During the multistep reaction the cumulative G-score (∑G) of the partially edited mRNAs progressively decreases ultimately dropping to zero in the fully edited mRNA (Fig. 3b). Similarly, the thermodynamic stabilities (expressed as ΔG/nt) decrease from −0.4 kcal/mol to −0.08 kcal/mol (Fig. 3a) and this trend is identical for all pan-edited transcripts in *T. brucei*, *L. tarentolae* and *T. cruzi*. Thus, the gRNA-directed insertion and deletion of U-residues resolves the individual GQ-elements in a stepwise fashion to generate less structured ORF’s. Eleven of the 17 gRNAs (65%) are required to resolve the 3 GQ’s of pre-edited CR4, which demonstrates that the U-insertion/deletion reaction not only serves to create a translatable ORF, it also functions to eliminate GQ-folds. This is further substantiated by the observation that the steady state abundance of gRNAs correlates with the position(s) of G-tracts in the different transcripts. The top 35% of gRNAs^15^ resolve 76% of all G-tracts between 2 and 8 G’s (2 ≤ G ≤ 8). This value is even more striking for G-runs of 4 ≤ G ≤ 8 (87%) or tracts of 5 ≤ G ≤ 8 (92%) (Table 1).

## Discussion

The majority of mitochondrial pre-mRNAs in the protozoan parasite *Trypanosoma brucei* are substrates of a U-insertion/U-deletion-type RNA editing reaction in order to be converted into translatable mRNAs. The reaction arguably represents one of the most bizarre RNA processing reactions since in several cases more than 50% of the final mRNA nucleotide content is a result of editing. Although the mechanism and the catalytic machinery of the biochemical process have been studied in detail^6^, a rational for the evolutionary driving force(s) and the biological necessity of editing are still missing. Here we report on a so far unrecognized structural feature of the pre-edited *T. brucei* transcripts. We discovered that specifically all pan-edited mitochondrial transcripts contain numerous clustered G-nt, which fold into multiple G-quadruplex structures. Altogether we experimentally identified 27 GQ-elements in eight of the nine pan-edited pre-mRNAs, with an average of 3 GQ’s per transcript. In addition, we uncovered that the different GQ-folds become resolved as the editing reaction proceeds from the 3′- to the 5′-ends of the transcripts, indicating a GQ-resolving quality of the process. This is further supported by the fact that the steady state abundance of guide (g)RNAs is positively correlated with the sequence positions of the different GQ-elements. Highly abundant gRNAs direct the editing reaction in predominantly GQ-fold containing RNA domains.

**Figure 4:**
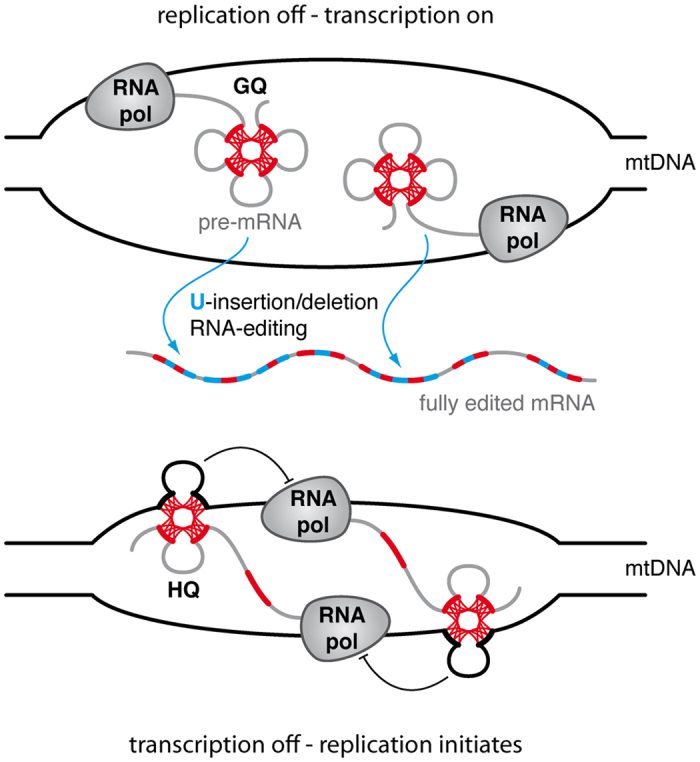
Switching between mitochondrial transcription and replication. Artistic rendering of the transcription-on/replication-off and transcription-off/replication-on phases of the *T. brucei* maxicircle DNA. GQ-elements in the synthesized transcripts define the transcription-on/replication-off state and require RNA editing to structurally resolve the different GQ-elements to generate translatable mRNAs (upper panel). The formation of HQ-folds between the nascent transcripts and the non-template strand(s) of the DNA maxicircle are characteristic for the transcription-off/replication-on situation (lower panel). GQ- and HQ-folds are shown as connected “leaf-like” structures. RNA pol: RNA polymerase.

Importantly, this so far unrecognized structural feature of all pan-edited *T. brucei* transcripts permits a new outlook onto the enigmatic evolutionary origin of the process^16^,^17^,^18^. If editing in part acts as a structural repair mechanism to generate GQ-free transcripts, then any potential function of GQ-folds must be located upstream of the processing reaction. GQ’s in RNAs have been identified as transcription termination and replication priming sites^19^. These functions are mediated through the formation of DNA/RNA hybrid G-quartet (HQ)-structures between the nascent transcript and the non-template DNA strand^20^,^21^,^22^. Thus, we hypothesize that the primary function and evolutionary advantage of the G-rich sequence motifs in the pan-edited transcripts lies in the formation of HQ-folds with the non-coding strand(s) of the maxicircle DNA. This generates multiple transcription termination sites and perhaps replication primers to assure the unperturbed replication of the mitochondrial genome. As was shown for human mitochondria, the separation of mitochondrial transcription and replication has the advantage that the two synthesizing machineries do not collide on the cicular DNA-template, which increases the processivity and fidelity of both processes^23^. This is even more important in African trypanosomes for two reasons: First, the organism has only one mitochondrion and second, a functional mitochondrion is absolutely required in the fly stage of the parasite life cycle. Cells with non-functional mitochondria or akinetoplastid parasites cannot survive in the insect as evidenced by the *T. brucei* subspecies *T. evansi* and *T. equiperdum*^24^. However, the selectional advantage of HQ-elements to favour mitochondrial replication creates a structural obstacle during all cell cycle stages requiring mitochondrial transcription. In the transcription-on state the G-rich sequences ultimately adopt the above identified GQ-folds, in addition to the fact that the cryptic pre-mRNAs lack substantial sequence information. Thus, the reduction in nucleotide complexity of mtDNA to favor the frequent formation of HQ-type transcriptional stops, inevitably requires a GQ-fold resolving and RNA sequence restoration activity in the transcription-on state. As a consequence we hypothesize that the two processes co-evolved and that HQ- and GQ-folds define mutually exclusive states of mitochondrial activity: HQ-elements identify the replication-on/transcription-off state and GQ-structures exemplify the replication-off/transcription-on situation, which requires RNA editing (Fig. 4).

The formation of HQ-folds is likely restricted to the kinetoplast S-phase, which precedes DNA-duplication of the nuclear genome^2^,^3^ Unfortunately, the molecular regulators that control the cell cycle timing of mitochondrial DNA-replication in *T. brucei* are only marginally understood^3^,^25^. For instance, it is not known whether protein factors homologous to the TEFM-protein in human mitochondria contribute to the replication/transcription switch^23^. However, a cell cycle-dependence of RNA editing has been suggested for *L. tarentolae*^26^ and we propose that the *T. brucei* editing accessory factor MRB1590 represents a mitochondrial GQ-interacting protein^27^. Additional support comes from the observation that the G-quadruplex binding compound diminazene aceturate^28^ inhibits mitochondrial DNA replication in *Trypanosoma cruzi*^29^. As predicted by our model this generates dyskinetoplastid parasite cells and reinforces a large body of earlier work demonstrating that kinetoplastid DNA and perhaps G-quadruplex structures represent suitable targets for a therapeutic intervention against trypanosomatid parasites^30^,^31^.

Our hypothesis is consistent with the observation that U-insertion/deletion-type RNA editing is restricted to the kinetoplastid protist lineage, an early diverging branch of the eukaryotes. Kinetoplastids are characterized by a single mitochondrion and strong alterations in the amount of mitochondrial involvement during their life cycle stages. Within this context, it is important to note that our hypothesis shares some aspects with the “RNA editing countering gene loss model” of Speijer^16^,^17^, in that it stresses the vulnerability of the mitochondrial system in these organisms. However, our model seems to have a stronger basis in observation and of course, these hypotheses are not mutually exclusive. On the other hand, our data are less compatible with a “constructive neutral evolution” scenario^18^ and instead suggest an interdependence of positive selection and coevolution as driving forces for RNA editing.

Lastly, we would like to stress that alternative interpretations including multiple functions of the different GQ-elements as well as inter-transcript quadruplexes can be envisaged. However, our structural data and the derived hypothesis clearly provide a novel framework for the evolutionary basis of RNA editing, which we hope will function as a stimulus to falsify the hypothesis.

## Methods

### Gene cloning and synthesis of pre-edited mRNAs

Mitochondrial genes encoding subunit 6 of the mitochondrial ATPase (A6), NADH dehyrogenase subunits 3, 5, 7, 8 and 9 (ND3, ND5, ND7, ND8, ND9), cytochrome C oxidase subunits 1 and 3 (CO1, CO3), C-rich regions 3 and 4 (CR3, CR4) and ribosomal protein S12 (RPS12) were PCR-amplified from *T. brucei* genomic DNA (strain Lister 427) followed by cloning into the SacI and KpnI restriction endonuclease sites of phagemid pBS SK^−^ II (Invitrogen). Sequences were verified by DNA sequencing and RNA transcripts were generated by run-off transcription using T7 RNA polymerase and linearized plasmids following standard protocols. Samples were DNaseI treated (37 °C, 15 min) followed by phenol extraction. Non-incorporated rNTP’s were removed by size exclusion chromatography and RNA transcripts were recovered by EtOH precipitation. RNAs were dissolved in 10 mM Tris/HCl pH 7.5, 1 mM EDTA (TE) and RNA concentrations were calculated from UV-light absorbancy measurements at 260 nm. The homogeneity and molecular sizes of the different RNAs was analyzed electrophoretically in 6% (w/v) urea-containing (8 M) polyacrylamide gels (Supplementary Fig. 1).

### Identification of potential GQ-forming sequences

*In silico* predictions of GQ-structures were performed for all *T. brucei* and *L. tarentolae* mitochondrial transcripts in their edited and unedited states. RNA sequences were retrieved from the U-insertion/deletion Edited-Sequence-Database (http://dna.kdna.ucla.edu/trypanosome/files/tbma-xi.html) and analyzed using QGRS Mapper^10^ and ViennaRNA 2.2.3^11^. QGRS Mapper predictions were performed using the low stringency constraints of ≥2 stacked G-tetrads and loop sizes between 0–36 nt. The likelihood of forming stable GQ-elements was quantified by calculating “G-score”-values^10^, which were summed up to yield cumulative (∑)G-scores per nt position. ViennaRNA-based predictions relied on a thermodynamic folding algorithm using the energy function: E [L, l] = a(L − 1) + b × ln(l − 2). L: number of G-tetrades (2 ≤ L ≤ 5); l: connecting loop length; a(37 °C): unpaired nucleotide penalty of −18 kcal/mol; b(37 °C): loop strain parameter of 12 kcal/mol. MFE-secondary structures and thermodynamic stabilities were extrapolated to 27 °C, which is the optimal growth temperature of editing-active, insect-stage trypanosomes.

### Experimental verification of GQ-folds and data analysis

The ability of G-clusters to adopt GQ-folds was experimentally verified at single nucleotide resolution using a differential reverse transcriptase (RT) stop assay at GQ-favouring (K^+^) and GQ-disfavouring (Na^+^, Li^+^) cation conditions. RT-stop reactions were performed using 1 pmol pre-edited transcripts and equimolar amounts of 5′-fluorescenctly labeled DNA-oligonucleotide primer molecules. After heat denaturation (95 °C, 1.5–2.5 min, 0.25xTE pH7.5) DNA-primers were annealed at 50–65 °C (7.5 min) followed by snap-cooling. Reverse transcription was performed by adding a concentrated reaction mix to yield final concentrations of 50 nM transcript, 50 nM DNA primers, 50 mM Tris/HCl pH8.3, 3 mM MgCl_2_, 5 mM dithiothreitol, 0.75 U/μL RiboLock^TM^ and 0.5 mM dNTP (each). Monovalent cation concentrations were varied between 1 mM and 75 mM until a sufficient readthrough was achieved (intra-mitochondrial K^+^-concentrations have been estimated in the range of 15 mM^32^). Some experiments required 0.75 mM Ni^2+^ as GQ-destabilizing cation^33^. Reaction mixes were incubated at 52 °C for 15–20 min at a SuperScriptIII^TM^ RT (ThermoFisher Scientific) concentration of 10 U/μL. RT-reactions were terminated by snap cooling followed by RNA hydrolysis in 400 mM NaOH (95 °C, 5 min). Resulting cDNAs were EtOH-precipitated, resuspended in Hi-Di™ formamide and analyzed by capillary electrophoresis (CE). Raw CE-traces were baseline adjusted and signal decay corrected using ShapeFinder^34^. Peak identities were determined by cDNA-sequencing followed by Gaussian peak integration. Pronounced stops were identified by boxplotting as peak integrals ≥1.25-fold the interquartile range. Remaining values were averaged and all peak integrals were divided by the average to normalize small peaks to ~1. GQ-induced stops were defined as stops showing a differential (Δ) response ≥2.5-fold the average peak integral in GQ-favouring (K^+^) *versus* GQ-disfavouring (Na^+^, Li^+^) conditions. Per transcript minimally 5 independent experiments were performed (mean *r* = 0.83). For a detailed analysis see Supplementary Fig. 5.

### RT-stop guided RNA 2D-structure prediction

RNA 2D-structures were generated with the help of ViennaRNA 2.2.3^11^ using the identified RT-stop positions to predict the folding and thermodynamic stability of the different GQ-elements. In some cases the effective loop length (l) was reduced by allowing long loops to adopt additional 2D-structure. GQ-sequence stretches with the lowest Gibbs free energies were forced into a single-stranded conformation during the subsequent 2D-structure prediction and were manually introduced to create RT-stop data refined minimal free energy (MFE) models. To estimate the Gibbs free energy of a given GQ-containing RNA structure all individual energy terms were summed up.

## Additional Information

**How to cite this article**: Leeder, W.-M. *et al*. Multiple G-quartet structures in pre-edited mRNAs suggest evolutionary driving force for RNA editing in trypanosomes. *Sci. Rep.* **6**, 29810; doi: 10.1038/srep29810 (2016).

## Supplementary Material

Supplementary Information

## Figures and Tables

**Table 1:** Correlation between gRNA abundance and their ability to resolve G-tracts.

resolved G-tracts/pre-mRNA	n-mer						
	2	3	4	5	6	7	8
A6	9	5	2	1	0	0	0
CR3	2	2	0	1	0	0	0
CR4	2	2	4	3	2	0	0
CO3	11	7	7	0	1	0	0
ND3	4	3	1	0	1	0	0
ND7	8	5	6	4	1	0	1
ND8	6	1	4	3	0	1	0
ND9	7	3	3	3	2	0	0
RPS12	4	1	1	0	1	0	0
G-tracts/pre-mRNA							
A6	21	6	5	1	1	0	0
CR3	5	4	0	1	0	0	0
CR4	8	2	6	4	2	0	0
CO3	31	12	9	1	1	0	0
ND3	6	4	3	1	1	0	0
ND7	30	11	10	5	1	0	1
ND8	14	1	5	3	0	1	0
ND9	13	7	4	3	2	0	0
RPS12	8	3	1	0	1	0	0
ΣG-tracts	136	50	43	19	9	1	1
Σresolved G-tracts	53	29	28	15	8	1	1
fraction resolved	0.39	0.58	0.65	0.79	0.89	1	1

On average, 76% of the G-tracts in the different pan-edited transcripts become resolved through the top 35% of gRNAs in the *T. brucei* gRNA-transcriptome^13^.
