# Supplementary material for: Multiple G-quartet structures in pre-edited mRNAs suggest evolutionary driving force for RNA editing in trypanosomes
Source: Sci Rep. 2016 Jul 20;6:29810. doi: 10.1038/srep29810 (PMC4951716; doi:10.1038/srep29810)
Supplement: Supplementary Information [file srep29810-s1.pdf]

Supplementary Figures/Tables

Multiple G-quartet structures in pre-edited mRNAs suggest evolutionary driving force for RNA editing in trypanosomes

W.-Matthias Leeder, Niklas F. C. Hummel, H. Ulrich Göringer

Molecular Genetics, Darmstadt University of Technology, Schnittspahnstraße 10, 64287 Darmstadt, Germany

Correspondence and requests for materials should be addressed to HUG (goringer@bio.tu-darmstadt.de).

Supplementary Figure 1. RNA characterization. Gelelectrophoretic characterization of the mitochondrial *T. brucei* CR3-, RPS12-, ND3-, A6-, CR4-, ND8-, ND9-, CO3- and ND7-transcripts used in this study. Electrophoresis was performed in 8M urea-containing 6% (w/v) polyacrylamide gels. M: molecular size marker. nt. nucleotides.

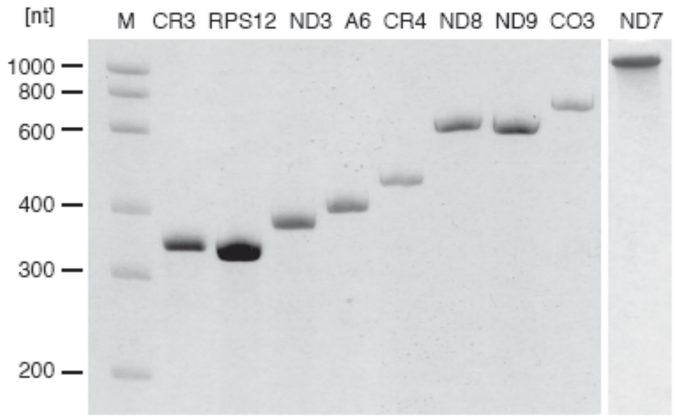

Supplementary Figure 2. Bioinformatic analysis of potential GQ-forming nucleotides. (a) QGRS Mapper analysis[10] of potential GQ-forming G-nt in the *T. brucei* mitochondrial genome. The figure shows a linear map of both strands of the entire *T. brucei* DNA maxicircle. Grey: ribosomal RNA genes (9S, 12S); red: pan-edited genes (ND8, ND7, CO3, A6, RPS12, ND3, CR4, ND9); green: marginally edited genes (Cyb, CO2, MURF II); blue: never-edited genes (ND4, ND5, CO1, ND1, ND2, MURF V). The height of the individual boxes indicates the relative G-content of the different ORF's (see Supplementary Table 1). The likelihood of forming GQ-motifs was caculated using the heuristic QGRS Mapper-based "G-score"-metric[10]. G-scores are non-negative integers and cumulative (Σ)G-score profiles are plotted above and below all relevant ORF's. Positions with low ΣG-scores are marked with arrows in red. (b) Blow-up of the 7kbp variable region of the *T. brucei* maxicircle.

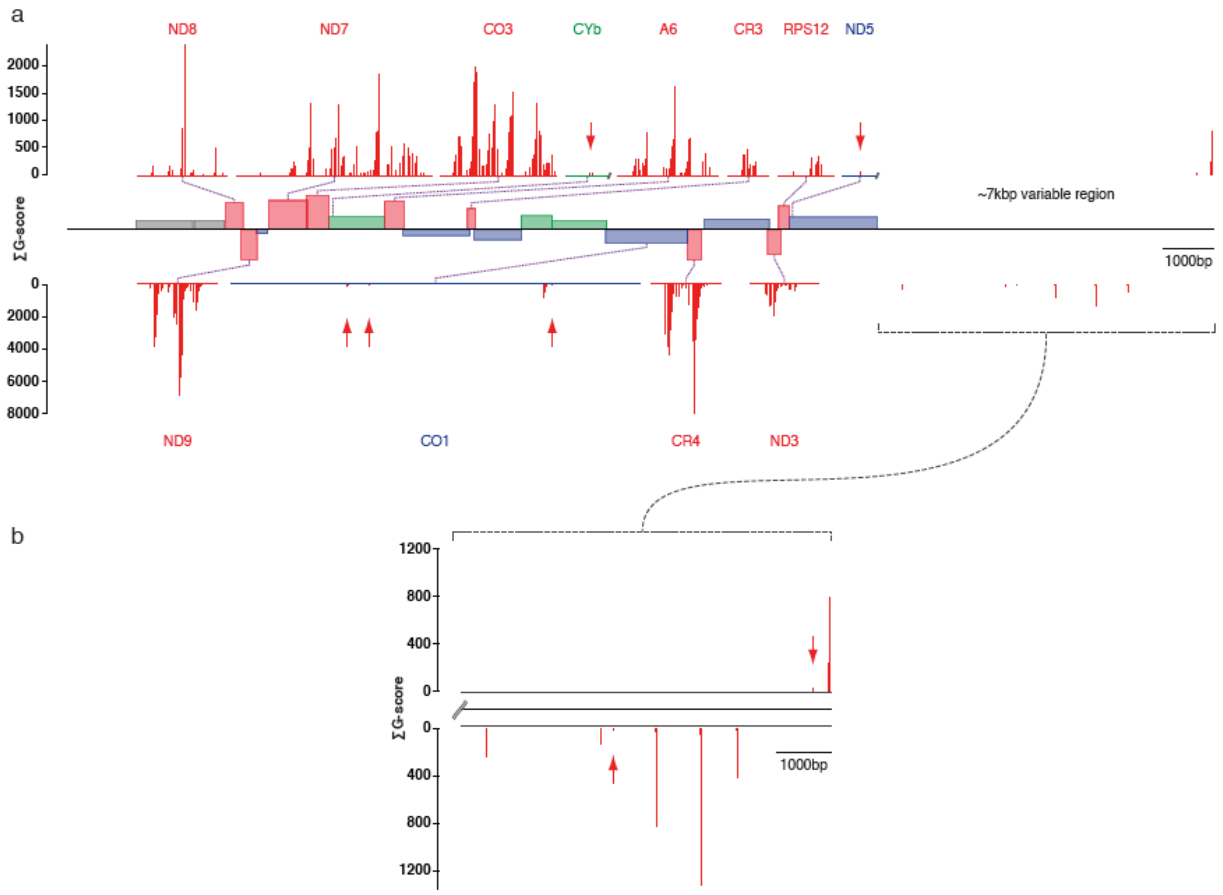

Supplementary Figure 3. Identification of potential GQ-forming nucleotides in the mitochondrial genome of *Leishmania tarentolae* and *Trypanosoma cruzi*. QGRS Mapper analysis[10] of potential GQ-forming G-nt in the coding region of the *L. tarentolae* (a) and *T. cruzi* (b) DNA maxicircles. The figure shows linear maps of both strands of the two maxicircles. Grey: ribosomal RNA genes (9S, 12S); red: pan-edited genes (ND8, CR3, RPS12, ND3, CR4, ND9); green: marginally edited genes (ND7, CO3, CYb, A6, CO2, MURF II); blue: never edited genes (ND4, ND5, CO1, ND1, ND2, MURF V). The height of the individual boxes indicates the fractional G-content of the different ORF's (y-axis on the left). Dashed horizontal line in black: average G-content of all transcripts (20%). Pan-edited genes are above the mean. The likelihood of forming a GQ-motif was caculated using the heuristic QGRS Mapper-based "G-score" metric[10]. Cumulative (Σ)G-score profiles are plotted above and below all relevant ORF's (y-axis on the right). Positions with low ΣG-scores are marked with arrows in red.

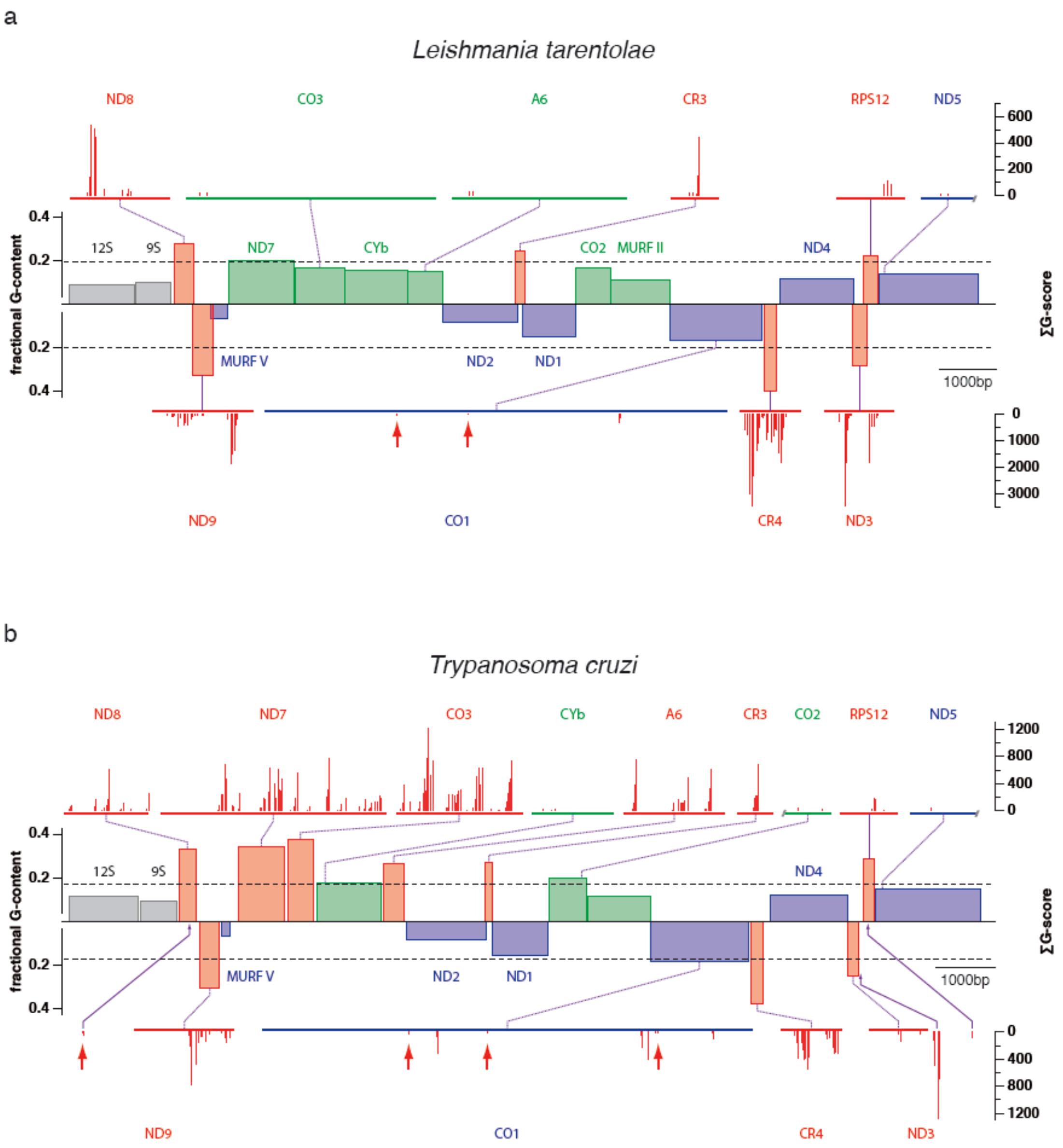

Supplementary Figure 4. RT-stop data map mutiple GQ-folds within a thermodynamic ensemble of RNA 2D-structures. (a) MFE-2D-structure of the pre-edited ND3 mRNA displaying two GQ-elements each containing three G-tetrades. (b) Corresponding RT-stop capillary electrophoresis (CE) profile at 75mM K^+. RFU: relative fluorescence unit. (c) The different peaks map to a sequence stretch between G_{140} and G_{202} containing four G-tracts (I to IV). (d) Arc representation of different GQ-elements that correlate with individual RT-stop signals (arrow heads in black) demonstrating the co-existence of GQ's of similar Gibbs free energies within an ensemble of RNA structures and in some cases of mutually exclusive GQ's. Red dots: G-nucleotides. ΔG's are in kcal/mol.

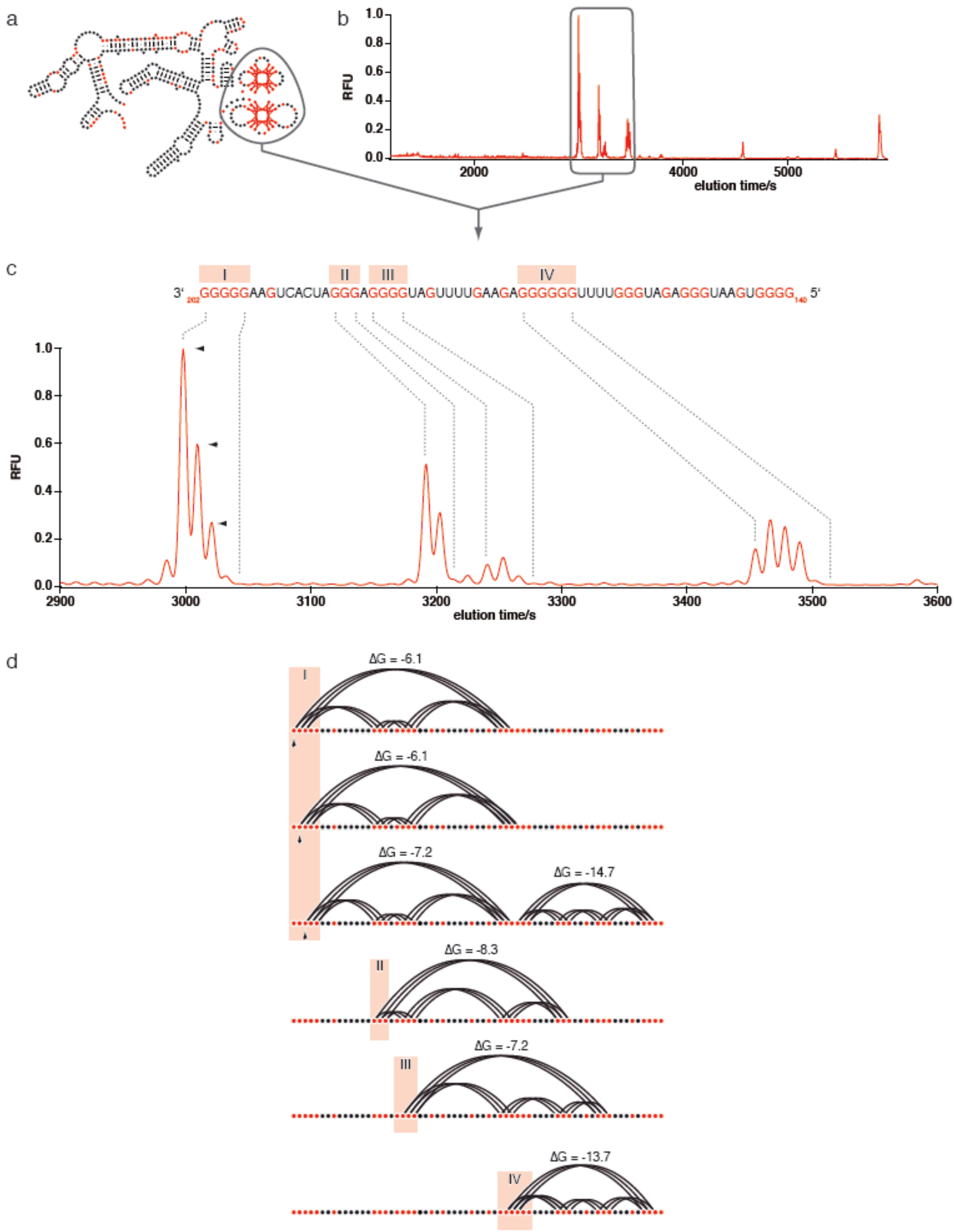

Supplementary Figure 5. Experimental variation of RT-stop experiments. (a) Normalized RT-stop data of the pre-edited CR3-transcript plotted as a function of the CR3-primary sequence. G-nt are highlighted in red. Error bars are SD. (b) Representative Pearson plots of three technical replicates of normalized RT-stop data. Blue: GQ-disfavoring (75mM Na^+) and red: GQ-favoring (75mM K^+) cation conditions. (c) Pearson (*r*) and Spearman's (ρ) correlation coefficients and average SD of all RT-stop data at GQ-disfavoring and GQ-favoring cation conditions. AU: arbitrary unit. nt: nucleotide.

a

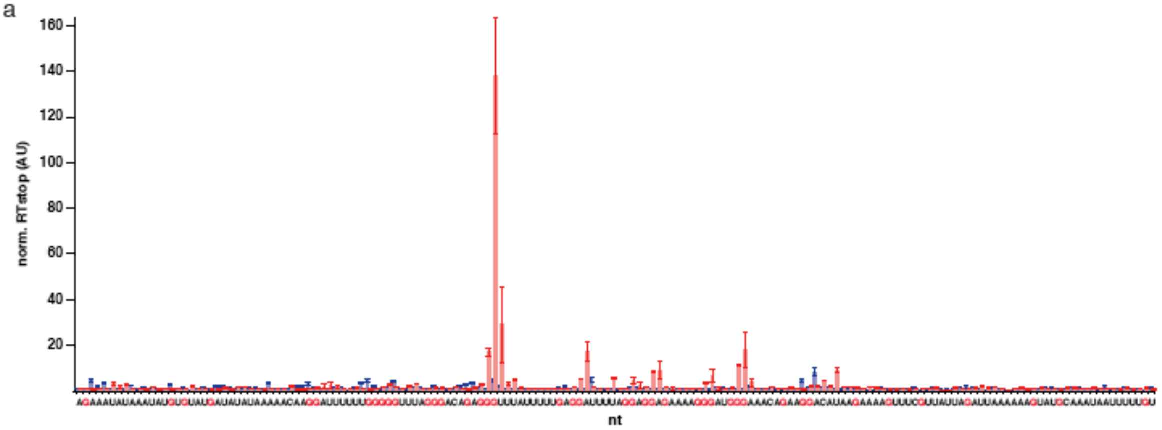

b

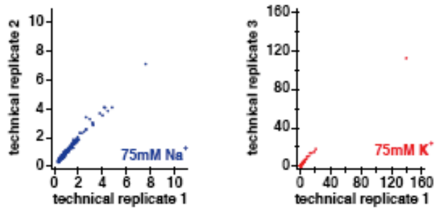

c

	GQ-disfavouring conditions	GQ-favouring conditions
Pearson (*r*)	0.97 ± 0.02	0.99 ± 0.01
Spearman (ρ)	0.94 ± 0.04	0.88 ± 0.03
average SD	0.11 ± 0.07	0.18 ± 0.13

Supplementary Table 1. Nucleotide propensities of all *T. brucei* mitochondrial transcripts. (a) pan-edited, (b) marginally edited and (c) never edited mRNAs. un: unedited; ed: edited. Pan-edited pre-mRNAs in their unedited state are purine-rich with R/Y ratios of $1.5 \leq R/Y \leq 2.7$. Red background: above average. Blue background: below average. (d) G-nucleotides are arranged in clusters. Pan-edited mRNAs contain roughly 2.5-times more G-tracts ($n \geq 2$) when compared to transcripts that are marginally or never edited. On average these clusters are only 10nt apart, which is 4-7-fold closer than in any other RNA analyzed.

a

pan-edited

mRNA	$A6_{un}$	$A6_{ed}$	$CR3_{un}$	$CR3_{ed}$	$CR4_{un}$	$CR4_{ed}$	$CO3_{un}$	$CO3_{ed}$	$RPS12_{un}$	$RPS12_{ed}$
length	401	820	164	300	283	568	463	970	221	325
#A	149	149	65	65	65	65	144	144	72	72
#G	137	137	42	42	107	107	195	195	64	64
#C	21	21	6	6	6	6	28	28	27	27
#U	94	513	51	187	105	390	96	603	58	162
R/Y	2.5	0.5	1.9	0.6	1.5	0.4	2.7	0.5	1.6	0.7
G's (%)	34	17	26	14	38	19	42	20	29	20

mRNA	$ND3_{un}$	$ND3_{ed}$	$ND7_{un}$	$ND7_{ed}$	$ND8_{un}$	$ND8_{ed}$	$ND9_{un}$	$ND9_{ed}$
length	268	465	783	1246	361	574	322	647
#A	87	87	244	244	100	100	99	99
#G	82	82	277	277	118	118	120	120
#C	20	20	90	90	44	44	25	25
#U	79	276	172	635	99	312	78	403
R/Y	1.7	0.6	2.0	0.7	1.5	0.6	2.1	0.5
G's (%)	31	18	35	22	33	21	37	19

b

marginally edited

mRNA	$CO2_{un}$	$CO2_{ed}$	CYb_{un}	CYb_{ed}	$MURF2_{un}$	$MURF2_{ed}$
length	632	636	1118	1152	1091	1113
#A	210	210	344	344	318	318
#G	107	107	184	184	123	123
#C	46	46	69	69	40	40
#U	269	273	521	555	610	632
R/Y	1.0	1.0	0.9	0.8	0.8	0.7
G's (%)	17	17	16	16	11	11

c

never edited

mRNA	CO1	ND1	ND4	ND5	MURF1	MURF5
length	1650	960	1314	1773	1343	234
#A	373	242	442	472	384	94
#G	286	120	167	267	101	11
#C	202	127	96	99	148	27
#U	789	471	609	935	710	102
R/Y	0.7	0.6	0.9	0.7	0.6	0.8
G's(%)	17	13	13	15	8	5

rRNA	9S	12S
length	611	1149
#A	249	462
#G	69	128
#C	35	67
#U	258	492
R/Y	1.1	1.1
G's(%)	11	11

d

mRNA	G's in G-tracts (%)	average distance between G-tracts (nt)
pan-edited$_{un}$	67.0	10
pan-edited$_{ed}$	25.4	40
marginally edited$_{un}$	29.7	49
marginally edited$_{ed}$	28.4	51
never edited	30.8	51
ribosomal RNAs	26.4	74
